# Venezuelan Equine Encephalitis Virus Capsid Implicated in Infection-Induced Cell Cycle Delay *in vitro*

**DOI:** 10.3389/fmicb.2018.03126

**Published:** 2018-12-18

**Authors:** Lindsay Lundberg, Jacque Fontenot, Shih-Chao Lin, Chelsea Pinkham, Brian D. Carey, Catherine E. Campbell, Kylene Kehn-Hall

**Affiliations:** ^1^National Center for Biodefense and Infectious Diseases, School of Systems Biology, George Mason University, Manassas, VA, United States; ^2^DCE Consulting, Vienna, VA, United States

**Keywords:** Venezuelan equine encephalitis virus, alphavirus, capsid, cell cycle, cyclin

## Abstract

Venezuelan equine encephalitis virus (VEEV) is a positive sense, single-stranded RNA virus and member of the New World alphaviruses. It causes a biphasic febrile illness that can be accompanied by central nervous system involvement and moderate morbidity in humans and severe mortality in equines. The virus has a history of weaponization, lacks FDA-approved therapeutics and vaccines in humans, and is considered a select agent. Like other RNA viruses, VEEV replicates in the cytoplasm of infected cells and eventually induces apoptosis. The capsid protein, which contains a nuclear localization and a nuclear export sequence, induces a shutdown of host transcription and nucleocytoplasmic trafficking. Here we show that infection with VEEV causes a dysregulation of cell cycling and a delay in the G_0_/G_1_ phase in Vero cells and U87MG astrocytes. Cells infected with VEEV encoding a capsid NLS mutant or treated with the capsid-importin α interaction inhibitor G281-1485 were partially rescued from this cell cycle dysregulation. Pathway analysis of previously published RNA-sequencing data from VEEV infected U87MG astrocytes identified alterations of canonical pathways involving cell cycle, checkpoint regulation, and proliferation. Multiple cyclins including cyclin D1, cyclin A2 and cyclin E2 and other regulators of the cell cycle were downregulated in infected cells in a capsid NLS dependent manner. Loss of Rb phosphorylation, which is a substrate for cyclin/cdk complexes was also observed. These data demonstrate the importance of capsid nuclear localization and/or importin α binding for inducing cell cycle arrest and transcriptional downregulation of key cell cycle regulators.

## Introduction

Venezuelan equine encephalitis virus (VEEV) is a positive sense, single-stranded, non-segmented RNA virus (Wagner and Hewlett, 2004). VEEV, along with the closely related eastern (EEEV) and western equine encephalitis viruses (WEEV), are part of the family *Togaviridae*, genus *Alphavirus*, and considered part of the “New World” clade owing to their geographic distribution and disease course (Strauss and Strauss, 1994). Due to ease of aerosolization, low infectious dose in humans, and the ability to grow to high titers in tissue culture, VEEV was weaponized by both the US and USSR as an incapacitating agent (Steele et al., 2007; Leitenberg et al., 2012). Thusly, VEEV is considered a select agent by the Centers for Disease Control and Prevention. There are no FDA-approved vaccines or therapeutics for use in humans, making VEEV an agent of concern for potential bioterrorist use (Sidwell and Smee, 2003). TC83 is a live attenuated vaccine strain, which has investigational new drug status and can be used for at risk personnel. TC83 was generated via passaging in guinea pig heart cells 83 times (Berge et al., 1961) and its attenuation is due to mutations in the 5′-non-coding region and the E2 glycoprotein (Kinney et al., 1993).

Naturally spread primarily by mosquitos, epizootic strains of VEEV causes a febrile-like illness in humans and high rates of mortality in equines. Though mortality is rare in humans, severe cases progress into central nervous system involvement and are accompanied by long-term sequelae including memory loss, insomnia, and personality change, especially in children (Steele et al., 2007; Steele and Twenhafel, 2010). Incubation is typically 2–5 days, and acute disease persists for 4–6 days; occasionally the disease becomes biphasic 4–8 days later with neurological and systemic involvement such as brain hemorrhaging and edema, necrotizing vasculitis, lymphocyte destruction, neutrophilic infiltration, and neuronal degradation (Weaver et al., 2004). There are many research gaps surrounding the process of VEEV neuroinvasion (especially from sites of peripheral inoculation), target cell populations, host/virus interactions, and the molecular impact of infection on host cell processes such as cell cycle, proliferation, antiviral responses, and apoptosis (Weaver et al., 2004; Steele et al., 2007; Reichert et al., 2009; Lundberg et al., 2017).

RNA viruses are known to moderate the host's cell cycle. Influenza A, a negative sense, segmented RNA virus, arrests cells in G_0_/G_1_; infected cells showed decreased retinoblastoma protein (Rb) phosphorylation and a decrease in other cell cycle modulators such as p21, cyclin E, and cyclin D1 (He et al., 2010). Rift Valley fever virus, a negative sense, segmented RNA virus, induces phosphorylation of DNA damage response proteins—ATM, Chk2, H2AX, and p53—in the absence of gross DNA damage and arrests cells in the S phase, which aids viral replication (Baer et al., 2012). The positive sense RNA coronavirus, infectious bronchitis virus, also downregulates cell cycle modulators such as cyclins D1 and D2, trapping infected cells in the G_2_/M phase (Dove et al., 2006). Another positive sense RNA virus, porcine reproductive and respiratory syndrome virus, downregulates cell cycle pathways on the transcriptional level, leading to cell cycle arrest at the S phase (Sun et al., 2014).

Actively dividing cells are subject to signaling cascades that moderate their progression through the cell cycle. Checkpoint modulators are typically switched “on” through phosphorylation, which in turn enables their own kinase activity. Cyclin-dependent kinases (CDKs) are typically thought of as the master regulators. They are usually constitutively expressed, but they have no activity until bound to their corresponding cyclin. Cyclins do not possess kinase activity, but their levels fluctuate corresponding to the phase of the cell cycle where they are needed. Cyclin D is expressed throughout the cell cycle, but is specifically needed for entry into the cell cycle. Cyclin E regulates the progression from G_1_ into S phase. Cyclin A levels begin to rise in S phase then plateau in G_2_. Cyclin B is necessary for progression from G_2_ into mitosis. Stressors such as nutrient deprivation or DNA damage stall or halt the cell cycle until the issue is remedied or the cell is signaled to undergo apoptosis [cell cycle reviewed extensively in (Graña and Reddy, 1995; Weinberg, 1995; Schafer, 1998)].

Here we show that infection with VEEV delays the cell cycle and disrupts regulation on a transcriptional level. In synchronized Vero cells, replicating virus induces a delay in the G_0_/G_1_ phase. The trafficking of the viral capsid protein is at least partially responsible for the delay, as cells infected with a viral strain coding for capsid with a mutated nuclear localization signal (NLS), referred to as VEEV TC83_Cm (Atasheva et al., 2010a; Lundberg et al., 2016), reentered the cell cycle sooner than cells infected with VEEV TC83. Cell cycle-related genes are largely downregulated in VEEV TC83-infected cell, but the phenotype is reversed with VEEV TC83-Cm.

## Materials and Methods

### Cell Culture

Vero and U87MG cells were maintained as previously described (Lundberg et al., 2013). To synchronize cell cycles in a population, DMEM without fetal bovine serum (FBS) for Vero cells or with low levels for U87MG cells (0.5–1%) was used to push cells into G_0_. Briefly, cells were seeded in complete media overnight, washed once with sterile PBS, then serum-free DMEM was added. Cells were starved for 72 h prior to infection as described below, then released using complete media.

### Viruses and Infections

VEEV-TC83 and VEEV-TC83_Cm viral stocks were produced as described previously (Lundberg et al., 2016) and utilized under BSL-2 conditions. VEEV-TrD working stocks were produced as described previously (Lundberg et al., 2016) and utilized under BSL-3 conditions. All work involving select agents is registered with the Centers for Disease Control and Prevention and conducted at George Mason University's Biomedical Research Laboratory, which is registered in accordance with federal select agent regulations.

To infect cells, virus was added to supplemented DMEM at a multiplicity (MOI) of 10 (VEEV-TC83) or 5 (VEEV-TrD), unless otherwise stated. Viral inoculum was added to the cells and incubated at 37°C for 1 h with rotation every 15 min. Viral inoculum was then removed, cells washed once with sterile PBS, and supplemented media added back to the cells, unless otherwise stated.

Crystal violet plaque assays determined viral titer as previously described (Lundberg et al., 2013).

### UV Inactivation

UV inactivation of VEEV TC83 was performed as described previously (de la Fuente et al., 2018). Briefly, viral supernatants were exposed to 120,000 μjoules five times, mixed, pooled, aliquoted, and stored at −80°C. Titers were determined by plaque assay and virus stocks considered inactivated if there were no plaques.

### Flow Cytometry

**Figure 1:**
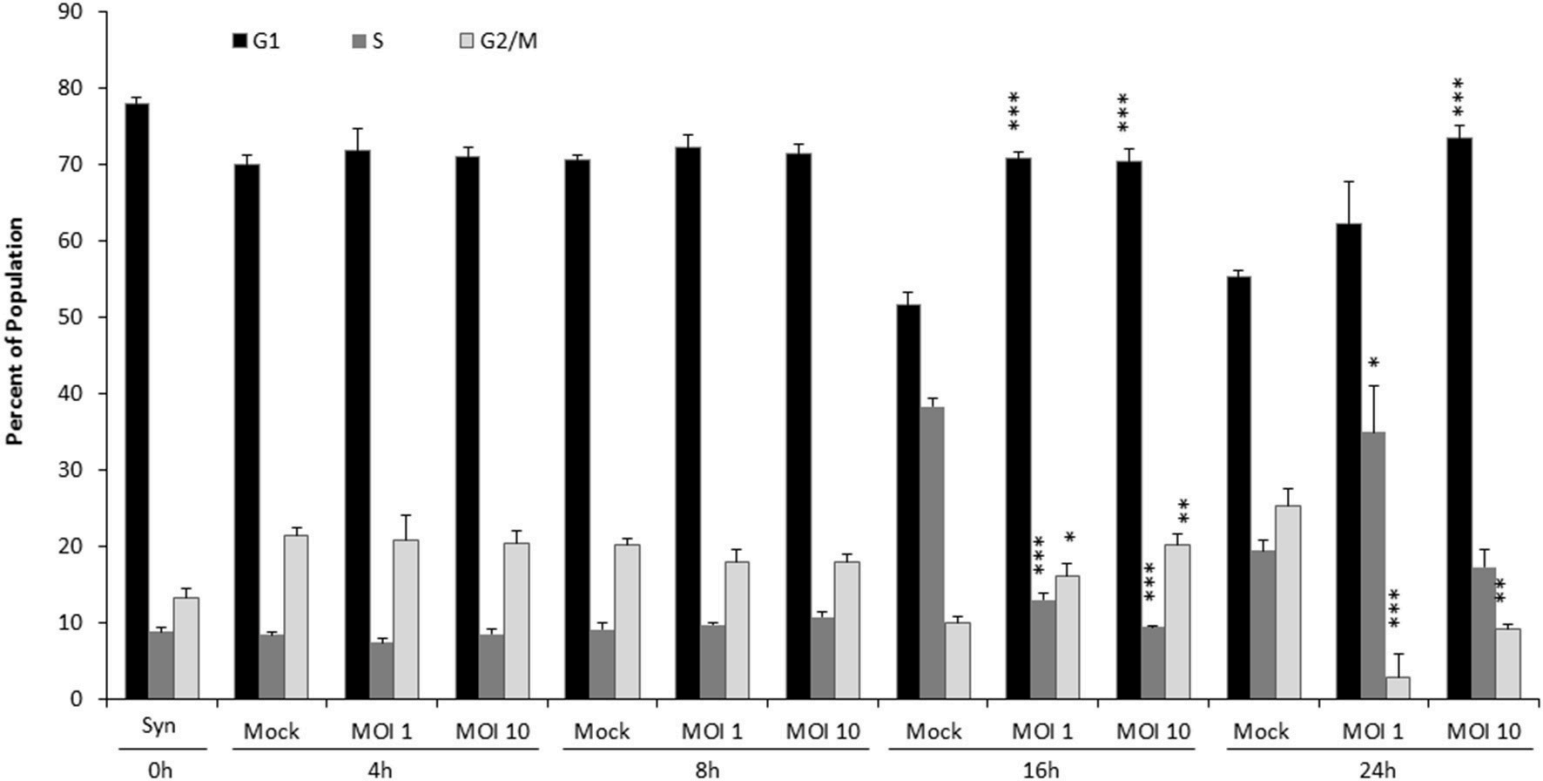
VEEV TC83 infection delays cell cycle in released, synchronized Vero cells. Vero cells were synchronized via serum-starvation for 72 h. Cells were then infected with TC83 (MOI 1 or 10) or mock-infected for 1 h then released in complete media containing serum. Cells were collected at 4, 8, 16, and 24 hpi, fixed and stained with PI then analyzed for cell cycle by flow cytometry. The average of three biological replicates is displayed. Statistical significance is calculated between mock and infected samples. ^*^p < 0.05, ^**^p < 0.005, ^***^p < 0.0005.

**Figure 2:**
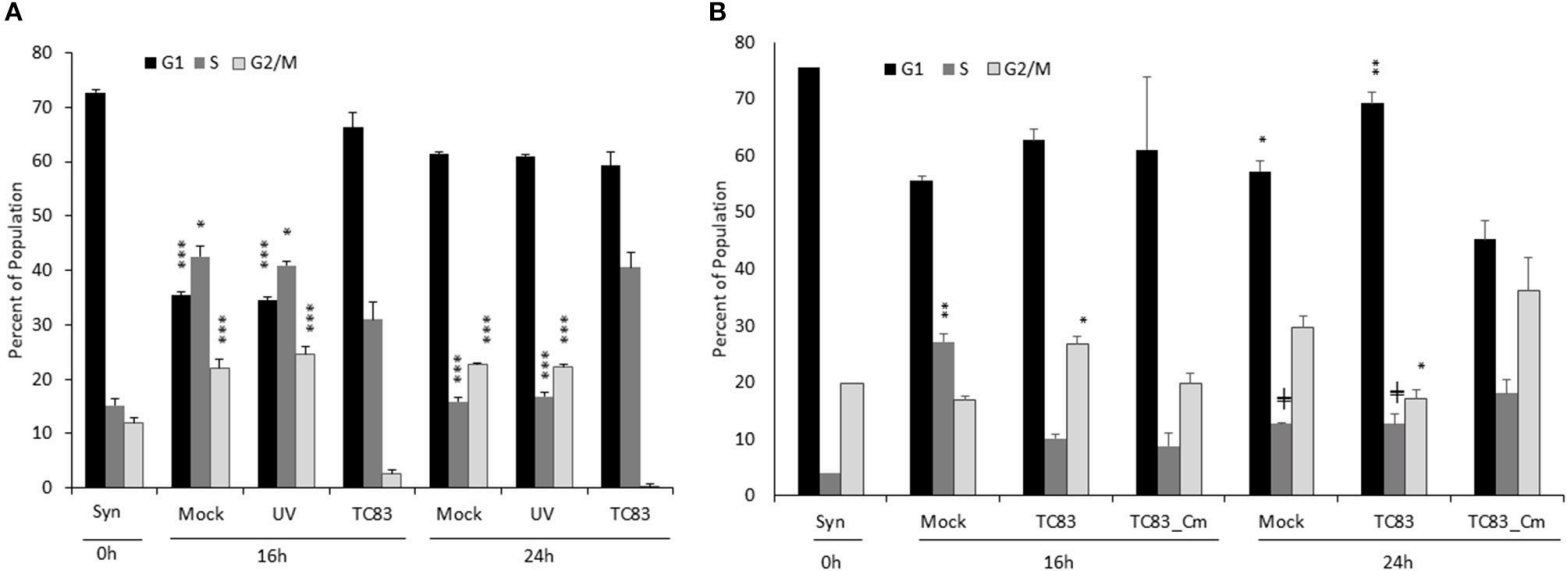
The cell cycle delay is partially dependent on replicating virus and capsid competent for nuclear import. **(A)** Vero cells were synchronized via serum-starvation for 72 h. Cells were then infected with TC83 (MOI 1), UV-inactivated TC83, or mock-infected for 1 h then released in complete media containing serum. Cells were collected at 16 and 24 hpi, fixed and stained with PI then analyzed for cell cycle by flow cytometry. The average of three biological replicates is displayed. **(B)** Similar to **(A)**, Vero cells were infected (MOI 10) with wild-type TC83, TC83_Cm, or mock infected then analyzed by flow cytometry. The average of three biological replicates is displayed, except for the 0 h samples which is *N* = 1. ^*^Statistical significance compared to mock-infected samples, ^+^Significance compared to TC83_Cm. ^+^*p* < 0.05, ^*^p < 0.01, ^**^p < 0.001, ^***^p < 0.0001.

Flow cytometry to determine population cell cycle phases was performed as described previously (Baer et al., 2012). Briefly, cells were trypsinized then washed before fixing with cold 70% ethanol. Cells were rehydrated in 1X PBS, spun down, then stained with propidium iodide (PI) staining solution. Cell cycle analysis was performed on an Accuri C6 flow cytometer running CFlow Plus (Figures 1, 2A) or FACSCalibur flow cytometer and analyzed with BD FACSDiva software. A minimum of 10,000 events were analyzed for each sample.

### RNA Sequencing and Ingenuity Pathway Analysis

Previously published RNA sequencing data (Baer et al., 2016a) was mined and analyzed using Ingenuity Pathway Analysis (IPA, Qiagen Bioinformatics; https://www.qiagenbioinformatics.com/products/ingenuity-pathway-analysis) to determine which cellular networks were altered at the transcriptional level. The raw sequencing data used for this analysis are publically available in the NCBI BioProject database under accession number PRJNA300864 (http://www.ncbi.nlm.nih.gov/bioproject/PRJNA300864). Fold changes and *p*-values were imported into IPA program and analysis performed using the Core Analysis function. Genes that changed more than 1.5X in infected cells, as compared to mock infected cells, and had *p* < 0.05 were used for downstream analysis. Canonical pathways altered after infection were displayed within IPA and manually mined to identify those associated with cell cycle.

### RNA Extraction and RT-qPCR

Infected cells were lysed and collected in Qiagen's RLT Buffer. RNA was isolated using Qiagen's RNeasy Mini Kit (74104) according to the manufacturer's directions. RNA from VEEV-TrD cells were converted to cDNA using the High Capacity RNA-to-cDNA kit (Applied Biosystems, 4387406) according to the manufacturer's protocol. qPCR for host genes was performed using TaqMan Gene Expression Master Mix (Applied Biosystems, 4369016). RNA isolated from VEEV-TC83 cells was assayed by RT-qPCR for host genes using the TaqMan RNA-to-C_T_ 1-Step Kit (Applied Biosystems, 4392938). Gene expression was assayed using the following TaqMan assays: HDAC9 (Hs01081558_m1), CDK6 (Hs01026371_m1), HDAC10 (Hs00368899_m1), CDK2 (Hs01548894_m1), CCNA2 (Hs00996788_m1), CCNG1 (Hs00171112_m1), CCNE2 (Hs00180319_m1), CDK1 (Hs00938777_m1), CCNB1 (Hs01030099_m1).

### Western Blot Analysis

Protein lysates production and western blotting were performed as described (Baer et al., 2012, 2016b). Blots were probed with anti-cyclin D1 (Cell signaling Cat#2978) anti-cyclin E2 (Cell Signaling Cat#4132), anti-cyclin A2 (Cell Signaling Cat#4656), anti-VEEV capsid (BEI Resources, NR 9403), and HRP-conjugated actin (catalog number ab49900-100, Abcam) antibodies.

### Statistical Analysis

Unless otherwise stated, all statistical analysis was calculated with the unpaired, two-tailed Student *T*-test using GraphPad's free online software, QuickCalcs.

## Results

### VEEV TC83 Infection Delays the Return to Cell Cycle of Synchronized Vero Cells

To analyze the effect of VEEV infection on cell cycle, Vero cells were serum-starved (SS) for 72 h, diverting the population to exit the cell cycle and enter G_0_. After synchronization, cells were infected with the live-attenuated vaccine strain of VEEV, TC83, for 1 h at a multiplicity of infection (MOI) of 1 or 10, washed with sterile PBS, then complete media replaced, releasing the cells back into the cell cycle. Propidium iodide (PI) was used to stain for DNA, and cell cycle progression measured using flow cytometry (Figure 1). Cells were collected immediately prior to infection as synchronized controls (Mock, 0 h), with 78% of cells in G_0_/G_1_, 9% in S phase and 13% in G_2_/M phase. G_1_, S, and G_2_/M proportions are the same for 4 and 8 hpi regardless of infection or MOI. By 16 hpi, mock-infected cells re-entered the cell cycle, as evidenced by the decrease in the G_1_ population and an increase in 38% of cells in S phase. In contrast at 16 hpi, VEEV infected cells (both MOI 1 and 10) displayed similar cell cycle profiles to cells at 0 h. By 24 hpi, cells infected at MOI 1 had also re-entered the cell, but a delay in G_1_ is still seen in cells infected at MOI 10; meanwhile a greater percentage of mock-infected cells have transitioned to G_2_/M, indicating a nearly complete return to a normal cell cycle. Collectively, these data demonstrate that infection with VEEV TC83 stalls cells at G_0_/G_1_ compared to mock-infected cells, and the effect is more pronounced with an increasing MOI.

### Viral Replication and Capsid's NLS Are Necessary for Cell Cycle Delay

To confirm that actively replicating virus was responsible for the delay, Vero cells were also exposed to UV-inactivated virus. As before, Vero cells were synchronized by SS for 72 h, mock-infected or infected with UV-inactivated or infectious TC83 (MOI 1), washed, then released in complete media. Populations were collected at 16 and 24 hpi and analyzed for cell cycle phases by flow cytometry and PI staining. Cells exposed to UV-inactivated virus were indistinguishable from mock-infected cells at both time points (Figure 2A). As demonstrated previously (Figure 1), synchronized cells infected with VEEV are slow to return to the cell cycle, indicating it is actively replicating virus that induces the delay and not merely the presence of viral proteins and RNA (Figure 2A).

The Frolova lab previously demonstrated that the VEEV capsid has both an NLS and NES and forms a tetrameric complex with the host karyopherins CRM1 and importin α/β1 (Atasheva et al., 2010a). This complex halts host nuclear import and downregulates transcription (Garmashova et al., 2007; Atasheva et al., 2008). Mutating the NLS alters capsid localization, retaining it primarily in the cytoplasm (Atasheva et al., 2010a; Lundberg et al., 2016); additionally, chemically inhibiting the interaction between capsid and importin α/β1 reduces viral titer and prolongs cell survival (Lundberg et al., 2013; Shechter et al., 2017; Thomas et al., 2018). Mutation of the NLS also prevents capsid from host cell transcriptional suppression (Atasheva et al., 2010b). We hypothesized that capsid's ability to interfere with nucleocytoplasmic trafficking and/or transcription contributes to the observed cell cycle delay. To test this hypothesis, we used a TC83 strain coding for a mutated NLS in the capsid (Cm) (Atasheva et al., 2010a; Lundberg et al., 2016). This mutated capsid lacks the ability to gain access to the nucleus (Supplementary Figure 1C) (Lundberg et al., 2016) and its replication kinetics are not different than parental TC83 (Supplementary Figure 1B) (Atasheva et al., 2010b). At 16 hpi, mock infected cells had started to return to cycling while TC83 and TC83_Cm infected cells were still delayed in G_1_, but by 24 hpi, S and G_2_/M populations in TC83_Cm infected cells had increased while TC83 infected cells were still delayed (Figure 2B). Representative histograms also provide visual demonstration of cell cycle dysregulation (Supplementary Figure 1A).

**Figure 3:**
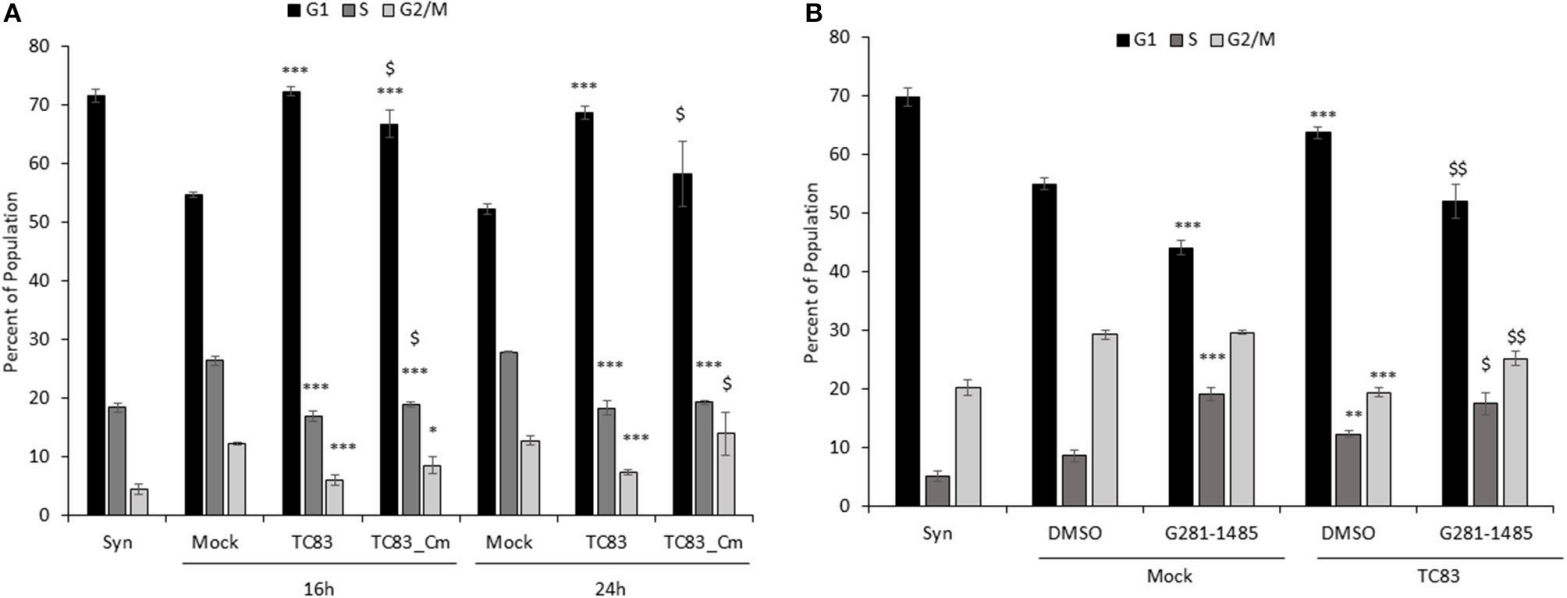
TC83 induces cell cycle delay in U87MG cells which can be relieved by a capsid-importin-α inhibitor. **(A)** U87MG cells were synchronized via serum-starvation (0.5% FBS) for 72 h. Cells were then infected (MOI 10) with wild-type TC83, TC83_Cm, or mock-infected for 1 h then released in complete media containing 10% FBS. Cells were collected at 16 and 24 hpi, fixed and stained with PI then analyzed for cell cycle by flow cytometry. The average of three biological replicates is displayed. ^*^Statistical significance compared to mock-infected samples, ^$^Significance compared to TC83 infected cells. ^*^p < 0.05, ^***^p < 0.001, ^$^*p* < 0.05 **(B)** Similar to **(A)**, U87MG cells were serum starved (0.1% FBS), treated with DMSO or G281-1485 (10 μM) for 1 h, infected (MOI 10) with wild-type TC83 or mock infected, and post-treated with DMSO or G281-145 in complete media containing 10% FBS. Cells were collected 24 h post-infection and analyzed by flow cytometry. The average of three biological replicates is displayed. ^*^Statistical significance compared to mock-DMSO samples, ^$^Significance compared to TC83-DMSO cells. ^**^p < 0.01, ^***^p < 0.001. ^$^*p* < 0.05, ^$$^*p* < 0.01.

The effect of VEEV on cell cycle progression was also assessed in U87MG astrocytes. Similar to what was observed in Vero cells, VEEV infection resulted in an accumulation of cells in G_0_/G_1_ phase (Figure 3A). Infection of U87MG cells with VEEV TC83_Cm displayed an intermediate phenotype and by 24 hpi more closely mirrored the cell cycle distribution of mock infected cells. Analysis of VEEV E2 positive cells revealed that the majority of the cell population was infected at 16 and 24 hpi (Supplementary Figure 2A). Further if cell cycle analysis was performed on only VEEV E2 positive cells for TC83 and TC83_Cm samples, cell cycle arrest was still observed (Supplementary Figure 2B). VEEV infection causes cell death and thus dying cells may confound the flow cytometry cell cycle analysis. To alleviate this issue, dead or dying cells were gated out during all flow cytometry experiments due to differences in forward and side scatter (Darzynkiewicz and Bedner, 2000). To further address this issue, the percent of viable cells following infection was assessed through Annexin V and PI staining, which allows the differentiation of necrotic and apoptotic cells (Supplementary Figure 3). After 72 h of serum starvation (Syn cells), U87MG cells displayed an average of 63% viable cells. By 16 hpi an increase in cell viability was observed in mock, TC83 and TC83_Cm infected cells. There were some differences in the necrotic and apoptotic cell populations at 16 hpi, but the total cell viability did not differ. By 24 hpi both TC83 and TC83_Cm infected cells displayed fewer viable cells with a corresponding increase in necrotic cells. These data indicate that there are some differences in cell viability at 24 hpi in both TC83 and TC83_Cm infected cells. However, the modest differences observed are not enough to explain the changes in cell cycle distribution. Collectively these results indicated that VEEV infected cells have an altered cell cycle progression profile.

To further explore the impact of capsid on cell cycle progression we employed the use of a compound, G281-1485, that was identified as an inhibitor of capsid-importin α interaction (Thomas et al., 2018). VEEV infected cells treated with G281-1485 displayed a cell cycle profile similar to mock infected cells, suggesting that disruption of capsid-importin α interaction relieves the cell cycle delay. Mock infected cells also revealed an altered cell cycle profile in the presence of G281-1485, which could be due to G281-1485 influencing other importin α cargo proteins. While G281-1485 can disrupt capsid-importin α interaction, it is not a selective inhibitor and therefore has the ability to impact other importin α cargo, as demonstrated by its ability to disrupt the SV40 large T antigen-importin α interaction (Thomas et al., 2018). Thus, capsid is at least partially responsible for the delay at G_1_and dysregulation of nuclear import and/or transcription is certainly a contributing factor to VEEV-induced cell cycle abnormalities.

### VEEV Dysregulates Cell Cycle at the RNA Level in Astrocytes

**Figure 4:**
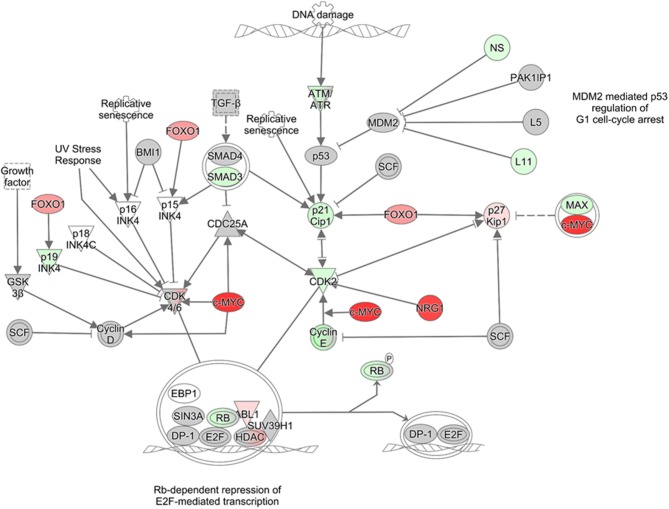
Pathway analysis graph of G1/S checkpoint. Gene expression changes in the G_1_/S checkpoint pathway generated with IPA. Gene expression values of U87MG cells infected with VEEV-TrD at an MOI 5 at 16 hpi (*N* = 3 biological replicates) as compared to mock infected cells. Red indicates upregulation and green downregulation of the given gene, and color intensity corresponds to relative difference in gene transcription compared to mock controls. Gray indicates genes that are in the dataset, but did not meet the fold-change or *p*-value threshold.

To determine potential mechanisms by which VEEV induces alterations in the cell cycle, we mined previously published (Baer et al., 2016a) RNA sequencing data generated from U87MG astrocytes infected with fully virulent VEEV-TrD (MOI 5, 16 hpi). Ingenuity Pathway Analysis (IPA) was used to determine canonical pathways associated with cell cycle, checkpoint regulation, and proliferation that were significantly altered following VEEV infection (Table 1). Compared to uninfected controls, many cell cycle pathways were downregulated (denoted by a negative z-score, the measure of correlation between relationship direction and gene expression). These changes were significant as shown by the –log(*p*-value), the results of the Fisher's exact test (the larger the value, the more significant). The ratio is the percentage of pathway coverage (but can be a biased value due to different pathway sizes). The last four columns indicate the number and percentage of genes downregulated, upregulated, that did not change, or did not overlap with the data set. “Cell Cycle: G_1_/S Checkpoint Regulation” in particular was predicated to be downregulated upon VEEV-TrD infection (z-score of −2.132), though a few individual genes within the pathway were upregulated, such as the cell cycle progression transcription factor c-MYC and the glucogenesis transcription factor FOXO1 (Figure 4). Both cyclin E and CDK2, which are responsible for phosphorylating Rb and allowing cells to pass through the restriction point in G_1_ (Weinberg, 1995; Lundberg and Weinberg, 1998), were downregulated (Table 2, Figure 4). Gene expression changes for individual cell cycle genes were also examined; for the most part, the cyclins and CDKs of the later stages of cell cycle—S, G_2_, and M—were downregulated at the mRNA level compared to mock infected controls (Table 2). Interestingly, several Class II histone deacetylases (HDACs), markers of chromatin condensation and cessation of transcription, were upregulated, as was the G_1_ kinase CDK6 (Table 2). These data are in agreement with our cell cycle analysis, indicating infected cells are not progressing through the cell cycle with a block at G_1_.

**Table 1:** Ingenuity canonical pathways.

**Ingenuity canonical pathways**	**-log (*p*-value)**	**Ratio**	**z-score**	**Downregulated**	**No change**	**Upregulated**	**No overlap with dataset**
ATM signaling	6.55	0.362	−1.4	42/80 (53%)	1/80 (1%)	31/80 (39%)	6/80 (8%)
Cell Cycle: G1/S checkpoint regulation	4.70	0.344	−2.132	28/64 (44%)	0/64 (0%)	29/64 (45%)	7/64 (11%)
GADD45 signaling	4.22	0.526	NaN	13/19 (68%)	0/19 (0%)	4/19 (21%)	2/19 (11%)
p53 signaling	3.88	0.270	0	44/111 (40%)	1/111 (1%)	43/111 (39%)	23/111 (21%)
Cell Cycle: G2/M DNA damage checkpoint regulation	2.80	0.306	1.069	29/49 (59%)	0/49 (0%)	15/49 (31%)	5/49 (10%)
Role of BRCA1 in DNA damage response	2.14	0.244	−2.138	44/78 (56%)	0/78 (0%)	31/78 (40%)	3/78 (4%)
Cyclins and cell cycle regulation	1.81	0.231	0	30/78 (38%)	0/78 (0%)	38/78 (49%)	10/78 (13%)
Role of CHK proteins in cell cycle checkpoint control	1.52	0.236	−2.121	29/55 (53%)	0/55 (0%)	24/55 (44%)	2/55 (4%)
Cell cycle regulation by BTG family proteins	0.47	0.171	−1	14/35 (40%)	0/35 (0%)	18/35 (51%)	3/35 (9%)

*Pathways selected for cell cycle regulation and analyzed by Ingenuity Pathway Analysis (IPA). RNA from VEEV-TrD infected (MOI 5) U87MG cells was collected at 16 hpi, sequenced, and compared to mock-infected controls. Depicted in the left-hand column is the canonical pathway. From left to right is the p-value of the right-tailed Fisher's exact test (the p-value is represented as the -log(p-value), and in this case, the larger the value, the more significant), ratio (how many genes in the changed in the pathway over the total number of genes in the pathway, or percentage of pathway coverage), z-score (predicted directionality; NaN = not a number, unable to predict directionality, where a value of more than 2 or <-2 is considered significant), and the number and percentage of genes in each category (upregulated, downregulated, no change, or no overlap with dataset)*.

**Table 2:** Up- and downregulated genes in VEEV-TrD infected U87MG cells at 16 hpi.

**Genes**	**Fold change (log_**2**_)**	***p*-value**
**VEEV-TRD VS MOCK INFECTED UPREGULATED GENES**		
HDAC9	5.085	0.0045
CDK6	4.932	0.00871
HDAC10	3.897	0.0348
**VEEV-TRD VS MOCK iNFECTED DOWNREGULATED GENES**		
CCNE2	−3.1	0.00646
CDK1	−2.725	0.0458
CCNG1	−2.179	0.0467
CDK2	−1.769	0.032
CCNA2	−1.65	0.000561
CCNB1	−1.594	0.00602

*Individual genes were selected from pathways identified through IPA in Table 1. Genes were either upregulated (top) or downregulated (down). The fold change is presented in log_2_ and the p-value is also noted*.

**Figure 5:**
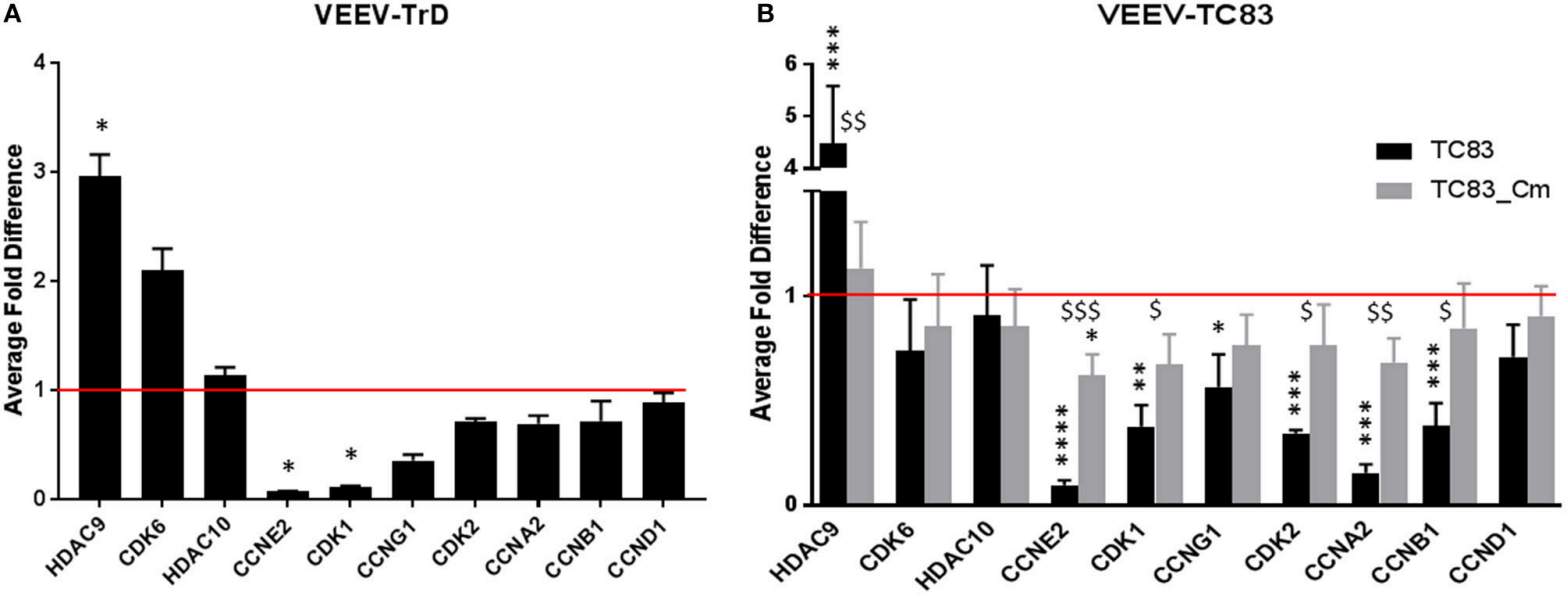
RT-qPCR confirmation of RNAseq cell cycle genes in VEEV infected cells. **(A)** U87MG cells were infected (MOI 5) with VEEV-TrD and then lysed at 16 hpi. After RNA extraction and conversion to cDNA, RT-qPCR was performed for the indicated genes and the fold difference compared to mock infected cells. The average of three biological replicates is displayed. **(B)** U87MG cells were infected (MOI 10) with TC83 or TC83_Cm then lysed at 16 hpi. After RNA extraction, RT-qPCR was performed for the indicated genes and the fold difference compared to mock infected cells. The average of three biological replicates is displayed. ^*^statistically significant differences from mock, ^$^statistically significant differences from TC83. ^*^p < 0.05, ^**^p < 0.01, ^***^p < 0.005, ^$^*p* < 0.05, ^$$^*p* < 0.01, ^$$$^*p* < 0.001.

Regulation of the individual genes were confirmed by reverse transcription quantitative PCR (RT-qPCR). As with the RNA sequencing data (Table 1), U87MG cells were mock-infected or infected with VEEV-TrD (MOI 5) then RNA extracted at 16 hpi. After converting the RNA to cDNA, the individual genes from Table 2 were analyzed against 18S RNA (Baer et al., 2016a) as the internal control. HDAC9 was the only gene whose upregulation compared to the mock control was statistically significant, but a general trend of upregulation of the other two genes was maintained (Figure 5A). For the predicted downregulated genes, only cyclin E2 (CCNE2) and CDK1 were statistically significant compared to mock cells, but again, there was a general trend of downregulation of the other cyclins and CDKs (Figure 5A, Table 2).

The previous flow cytometry data indicated that cells infected with TC83_Cm returned to the cell cycle quicker than cells infected with TC83 (Figure 2B). In addition, capsid is known to downregulate host transcription and TC83_Cm loses this ability (Atasheva et al., 2010b). Based on those results, we wanted to see what affect TC83_Cm would have on cell cycle genes on the RNA level. U87MG cells were mock-infected, infected with TC83, or infected with TC83_Cm (MOI 10). At 16 hpi, RNA was extracted and cell cycle genes (Table 2) were analyzed by RT-qPCR. Once again, HDAC9 was significantly upregulated, even in cells infected with the vaccine strain, but its levels in cells infected with TC83_Cm more closely resembled mock infected cells (Figure 5B). The other two predicted upregulated genes, CDK6 and HDAC10 were not altered in either TC83 or TC83_Cm infected cells. However, all but one cell cycle gene predicted to be downregulated were significantly down compared in TC83 infected cells and less depressed when infected with TC83_Cm (Figure 5B). Because TC83_Cm has a mutated NLS and remains in the cytoplasm, this indicates capsid is partially responsible for the dysregulation of the cell cycle related genes upon VEEV infection, and that its interaction with host karyopherins is also a contributing factor.

To discount differences in viral replication that may contribute to previous results, U87MG cells were infected (MOI 10) with TC83 or TC83_Cm. Supernatants in triplicate were collected at 4, 8, 16, and 24 hpi, and titers were determined by plaque assay (Supplementary Figure 1B). Though there is a small difference in titers between the two viruses at 16 hpi, the overall rate of replication across timepoints appears to be the same for the viruses. This indicates that TC83_Cm, though virulence is reduced in mice (Atasheva et al., 2015), is not replication deficient compared to TC83 in U87MG cells, as has been confirmed in other cell lines (Atasheva et al., 2015; Lundberg et al., 2016), and that differences in host transcriptional expression between the two viruses is owing to the mutated NLS.

### Cyclin Protein Expression and Rb Phosphorylation Are Decreased Following VEEV Infection

**Figure 6:**
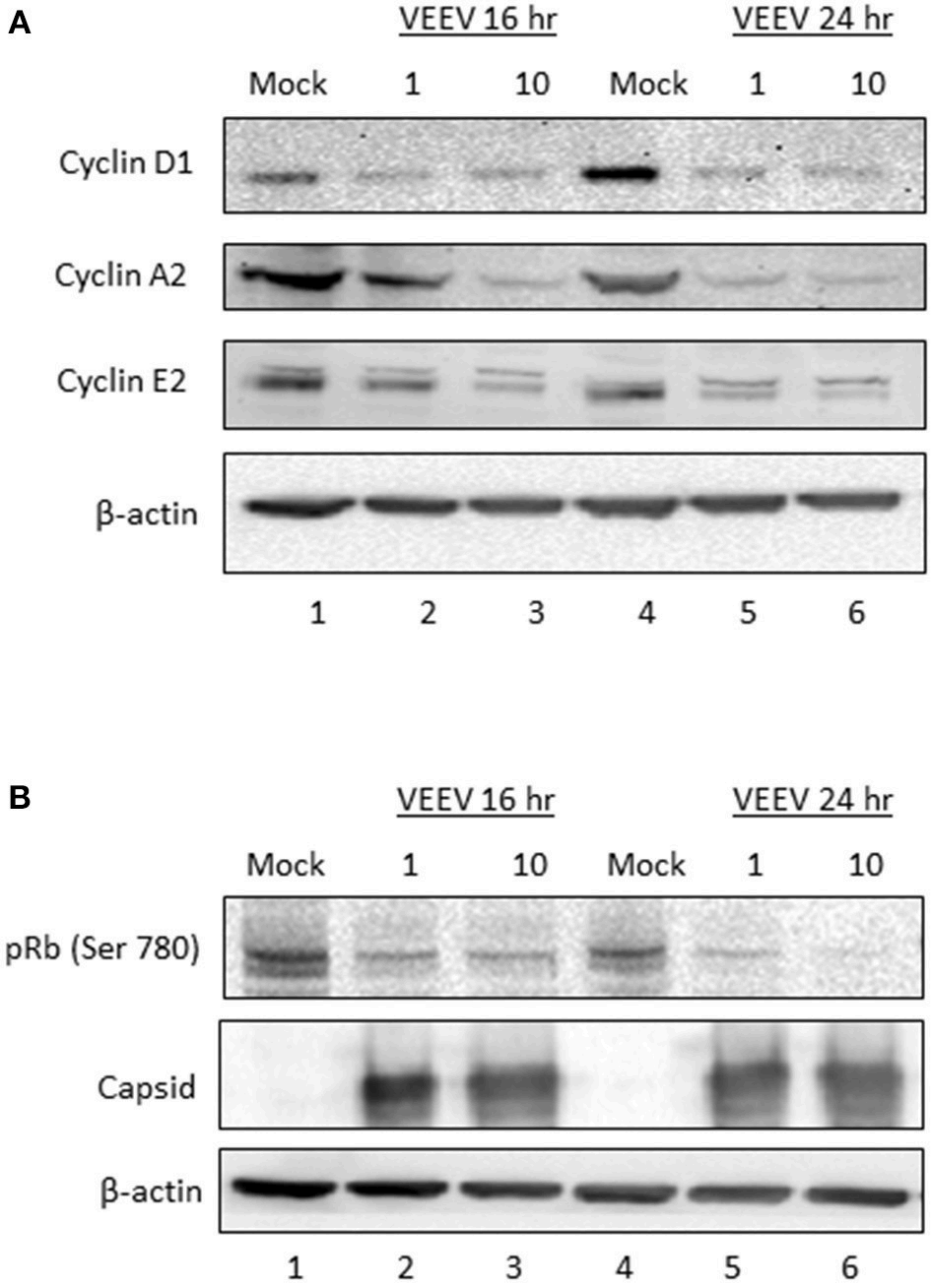
Cyclin protein expression and Rb phosphorylation are decreased following VEEV infection. **(A)** U87MG cells were synchronized via serum-starvation for 72 h. Cells were then infected (MOI 10) with VEEV TC83 or mock-infected for 1 h and then released in complete media containing 10% FBS. Cells were collected at 16 and 24 hpi for western blot analysis of cyclin D1, E2, A1 or actin expression. **(B)** Western blot analysis for phospho-Rb, capsid and actin levels in VEEV TC83 or mock infected U87MG cells.

To further explore the impact of VEEV infection on cell cycle regulation, cyclin D1, E2, and A2 protein expression was assessed via western blot analysis. Both cyclin E2 and A2 protein levels were decreased following VEEV infection at 16 and 24 hpi (Figure 6A), which is consistent with the observed decrease in mRNA expression (Figure 5B). Changes in cyclin E2 and A2 levels were MOI dependent at 16 hpi. Surprisingly cyclin D1 expression was also dramatically decreased in VEEV infected cells at 16 and 24 hpi (Figure 6A) even though cyclin D1 mRNA levels were not decreased (Figure 5B). Rb is a substrate of cyclin D and E complexes, with phosphorylation releasing the transcription factor E2F and allowing transcription of many cell cycle regulated transcripts (Vermeulen et al., 2003). Assessment of Rb phosphorylation at Ser780, which is a residue primarily phosphorylated by cyclin D1 (Kitagawa et al., 1996), revealed a loss of Rb phosphorylation following VEEV infection (Figure 6B). These results indicate that cyclin D1, E2, and A2 protein expression and Rb phosphorylation are reduced following VEEV infection, corresponding to the observed VEEV induced cell cycle alterations.

## Discussion

VEEV infection delays the cell cycle and disrupts host transcription, translation, and nucleocytoplasmic trafficking, which in turns results in aberrant cell cycling. In synchronized Vero and U87MG cells, replicating virus induces a delay in the G_0_/G_1_ phase (Figures 1, 2A, 3A). The association of the viral capsid protein with importin α is at least partially responsible for the delay, as cells infected with a viral strain (TC83_Cm) coding for capsid with a mutated NLS (Atasheva et al., 2010a; Lundberg et al., 2016) and treatment of cells with a capsid-importin α inhibitor reentered the cell cycle faster than cells infected with TC83 (Figures 2B, 3A,B). As nsP2 is known to induce host translational shutoff (Bhalla et al., 2016), it not surprising that mutation of capsid alone is incapable of completely correcting the observed cell cycle arrest. Another possible contributing factor is the induction of the unfolded protein response (UPR) following VEEV infection (Baer et al., 2016a). UPR induction is triggered by the sensing of improperly folded proteins and leads to inhibition of translation and potentially apoptosis (Walter and Ron, 2011). During viral infections a high burden is placed on the endoplasmic reticulum (ER) to fold the enormous amount of viral proteins (typically glycoproteins) being produced. VEEV infection induces the protein kinase RNA-like ER kinase (PERK) arm of the UPR, with corresponding increases in ATF4 and CHOP expression (Baer et al., 2016a). Interestingly, UPR activation via tunicamycin treatment results in decreased cyclin D1 protein translation and inhibition of cell cycle progression (Brewer et al., 1999). These findings are consistent with our data showing that VEEV infected cells have decreased cyclin D1 protein levels, but not mRNA levels. It is likely a combination of capsid induced transcriptional inhibition and translation (nSP2 and/or UPR mediated) suppression that alters the cell cycle following VEEV infection.

Cell cycle-related pathways and genes are mostly downregulated in VEEV-TrD (Figure 5A) and TC83-infected U87MG astrocytes, but the phenotype is not as pronounced when cells are infected with TC83_Cm (Figure 5B). Additionally, transcription of later stage cyclins is prominently downregulated, further suggesting that VEEV infected cells are stuck in an early phase, G_0_ or G_1_. From this perspective, VEEV infection may induce a G_0_/G_1_ delay, or “stutter” that is not as profound in TC83_Cm-infected cells and is also not a complete shutdown of the cell cycle (at least at lower MOIs). Further compounding the issue is the observation that cyclin D1 protein levels are dramatically decreased and Rb phosphorylation suppressed at 16 and 24 hpi. These data further support the model whereby cells that are forced into G_0_ via serum starvation are severely inhibited from reentering the cell cycle.

The inability to reenter the cell cycle could have profound implications for astrocyte function following VEEV infection. Once a controversial *in vitro* model, astrocytes have been shown to support VEEV infection *in vivo* in mouse models (Peng et al., 2013; Cain et al., 2017), though the major target cells in humans have yet to be identified (de la Monte et al., 1985; Ludlow et al., 2016) owing to the rarity of autopsy materials (Weaver et al., 2004). Astrocytes collect and distribute energy substrates and neurotransmitters in addition to defending and supporting neurons and contributing to the brain architecture and environment (Kettenmann, 2011). While astrocytes are typically quiescent cells, they have the capability to enter the cell cycle in response to injury (Buffo et al., 2008; Wang et al., 2009). Reactive astrogliosis is an increase in astrocyte numbers, which can be induced via multiple pathological conditions including trauma, infection, ischemic damage, neuroinflammation, or neurodegeneration (Pekny and Pekna, 2014). Between 1 and 50% of reactive astrocytes in various brain injuries are proliferating (Wang et al., 2009). Astrogliosis can be viewed as beneficial limiting tissue damage and restoring CNS homeostasis, but prolonged astrogliosis can lead to negative consequences including glial scar formation and inhibition of axon regeneration (Pekny and Pekna, 2014). Astrogliosis as indicated by increased GFAP staining, a marker of astrocyte proliferation, and apoptosis were observed in VEEV infected mice (Schoneboom et al., 2000). Both astrogliosis and neuronal apoptosis were positively correlated with pathogenesis. Astrogliosis was found in regions where VEEV antigen was present as well as in regions without detectable VEEV antigen, indicating it may be induced directly or indirectly by VEEV infection. Therefore, while astrogliosis does occur during VEEV infection *in vivo*, whether VEEV infection of astrocytes influences astrogliosis and cell cycle progression *in vivo* has not been determined. In addition, the long-term impact of astrogliosis is unknown, which is especially important to determine as VEEV infection in humans leads to neurological sequelae in 4–14% of VEEV survivors (Ronca et al., 2016). Based on our data, we speculated that astrogliosis would be inhibited in VEEV infected cells, as these cells would be severely limited in their ability to reenter the cell cycle. In contrast, VEEV infection of actively replicating astrocytes should lead to cell cycle arrest. In either case, these events would culminate in cell death in the majority of cells as VEEV infection induces cell death in a capsid dependent manner (Atasheva et al., 2010b). While a block in nucleocytoplasmic trafficking has been implicated in VEEV capsid induced cell death (Atasheva et al., 2010b), additional pathways altered prior to death have yet to be described. Our data demonstrate that VEEV infected cells are arrested in G1 with corresponding decreases in cyclin RNA and protein levels in a capsid dependent manner. These findings provide molecular insight into host pathway alterations that are triggered following VEEV infection, which may contribute to apoptosis.

**Figure 7:**
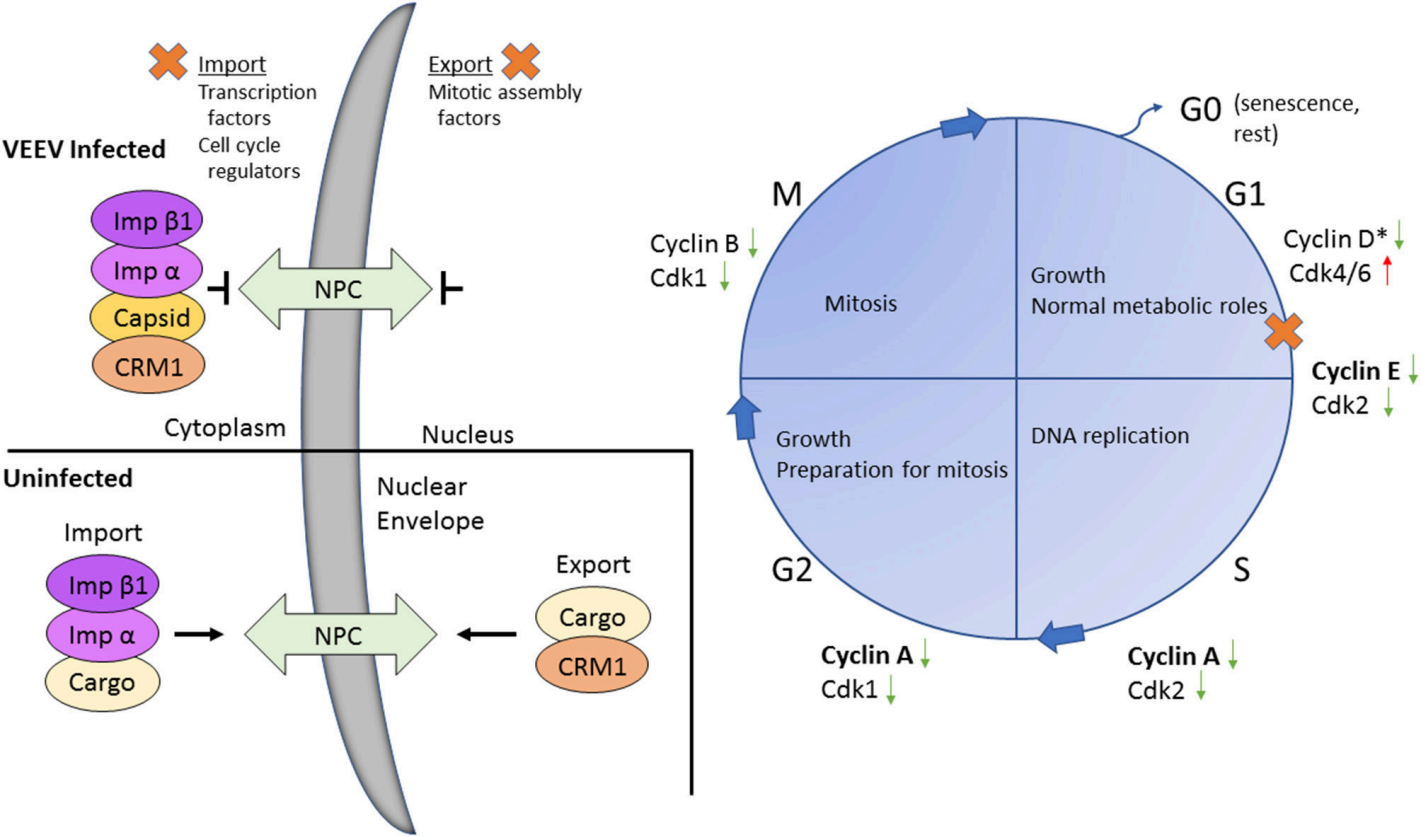
Model of VEEV infection induced transcriptional dysregulation leading to cell cycle delay. Upon VEEV infection, capsid blocks nucleocytoplasmic trafficking through forming a tetrameric complex with CRM1, importin α and importin β. This block results in transcriptional suppression, including an overall decrease in cell cycling associated transcripts, inducing a delay primarily at G_0_/G_1_. Nucleocytoplasmic trafficking is intact in uninfected cells. Proteins affected by the block in nucleocytoplasmic trafficking were not determined in this study but are hypothesized to be transcription factors, cell cycle regulators and/or mitotic assembly factors. The specific cyclin and cdk proteins affected following VEEV infection are indicated. Bolded cyclins (cyclin E and A) were decreased at both the mRNA and protein level, whereas expression of the unbolded cyclins and cdks were only assessed at the mRNA level. ^*^Cyclin D mRNA expression was not altered but protein levels were decreased.

Our data coupled with other previous findings led us to our model (Figure 7) where in VEEV infected cells, capsid binds to the host karyopherins CRM1 and importin α/β1, blocking the nuclear pore complex and shutting down host transcription. Transcriptional suppression is directly correlated with the block in nucleocytoplasmic trafficking although the exact mechanism is unknown (Garmashova et al., 2007; Lundberg et al., 2017). Capsid mediates transcriptional suppression of cyclins (A, E, G, and B) and cdks (cdk1 and cdk2), due at least in part to the depletion of host cell import and export proteins (Figure 7). Cyclin D1 protein expression and Rb phosphorylation (not shown on the model) are also impacted, which contribute to VEEV induced cell cycle arrest. Cyclin D1 expression is necessary for entry into G1 whereas cyclin E2 regulate the restriction point (G1/S checkpoint), which is the point in which the cell is committed to undergoing DNA replication (Vermeulen et al., 2003). Cyclin A expression is needed during S phase to facilitate DNA replication (Vermeulen et al., 2003). Therefore, decreased expression of all three of these cyclins severely limits the ability of VEEV infected cells to advance through the cell cycle. We hypothesize that transcriptional suppression of cyclins and cdk transcripts and the cell cycle delay could be influenced by this nucleocytoplasmic trafficking block, including reduced nuclear import of transcription factors or cell cycle regulators or decreased nuclear import of mitotic assembly factors. Future studies will explore these hypotheses and the importance of cell cycle alterations for VEEV pathogenesis.

## Author Contributions

LL, JF, and KK-H conceived and designed the experiments. LL, JF, CP, BC, and S-CL performed the experiments. LL, JF, CC, and KK-H analyzed the data. LL and KK-H wrote the paper.

### Conflict of Interest Statement

The authors declare that the research was conducted in the absence of any commercial or financial relationships that could be construed as a potential conflict of interest.
