# Supplementary material for: Venezuelan Equine Encephalitis Virus Capsid Implicated in Infection-Induced Cell Cycle Delay in vitro
Source: Front Microbiol. 2018 Dec 18;9:3126. doi: 10.3389/fmicb.2018.03126 (PMC6315117; doi:10.3389/fmicb.2018.03126)
Supplement: Supplementary file 1 [file Data_Sheet_1.PDF]

Supplementary Material

Venezuelan Equine Encephalitis Virus Capsid Implicated in Infection-Induced Cell Cycle Delay in vitro

Lindsay Lundberg[1], Jacque Fontenot[1], Shih-Chao Lin[1], Chelsea Pinkham[1], Brian Carey[1], Catherine Campbell[2], Kylene Kehn-Hall[1#]

# Correspondence: Kylene Kehn-Hall: kkehnhal@gmu.edu

1 Supplementary Figures and Tables

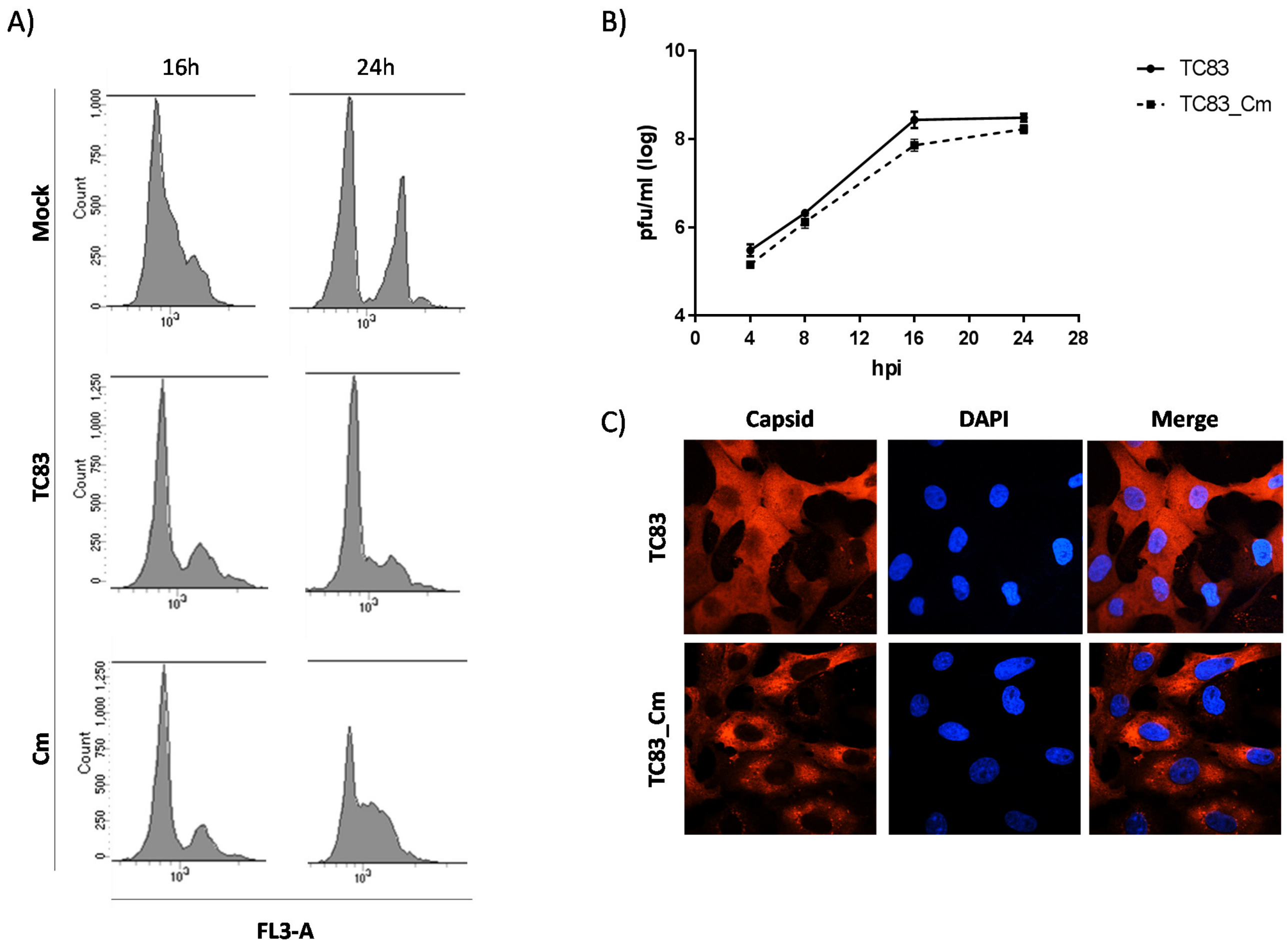

Supplementary Figure 1. (A) Histograms correlating to Figure 2B. Briefly, Vero cells were synchronized, infected (MOI 10) with TC83, TC83_Cm, or mock-infected for 1h, released, then collected at 16 (left) and 24 (right) hpi and analyzed for cell cycle by flow cytometry. (B) U87MG cells were infected (MOI 10) with TC83, TC83_Cm, or mock-infected for 1 h, washed with sterile

1X PBS, complete media replaced, then supernatants collected at 4, 8, 16, and 24 hpi. To determine titers, supernatants were serially diluted and BHK-J used for crystal violet plaque assays as previously described. (C) Vero cells were infected with TC83 or TC83_Cm for assessment of capsid expression and localization via confocal microscopy. At 16 hours hpi, cells were fixed and probed for capsid (red) and DAPI stained (blue).

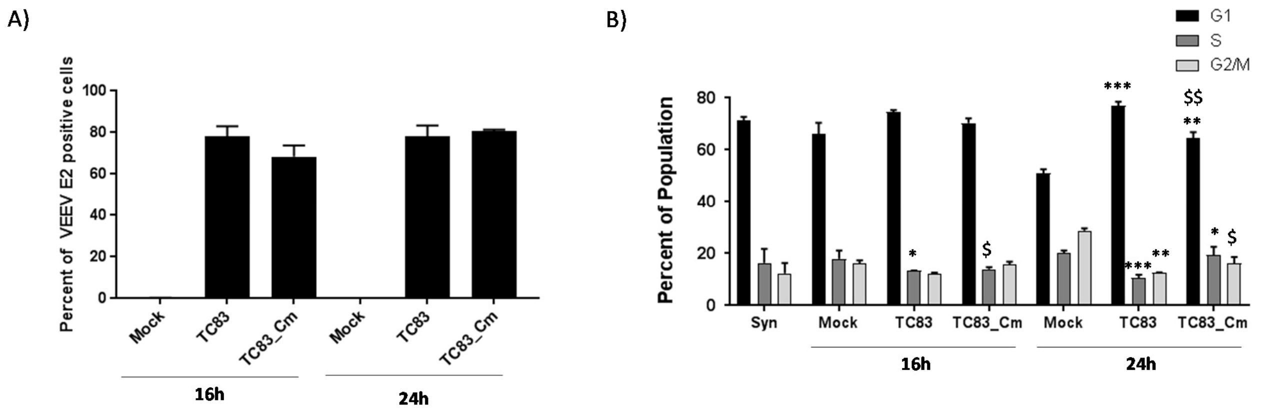

Supplementary Figure 2. U87MG cells were synchronized via serum-starvation (0.5% FBS) for 72 h. Cells were then infected (MOI 10) with wild-type TC83, TC83_Cm, or mock-infected for 1 h and then released in complete media containing 10% FBS. Cells were collected at 16 and 24 hpi, fixed and stained with PI. Cells were also incubated with anti-VEEV E2 primary antibody and anti-mouse IgG conjugated with AF488 secondary antibody to enable analysis of VEEV infected cells. Panel A displays the percent of VEEV E2 positive cells for the entire analyzed population. Panel B displays the cell cycle analysis. For TC83 and TC83_Cm samples, cell cycle analysis was performed on only E2 positive cells. The average of three biological replicates is displayed.* is statistical significance compared to mock-infected samples, $ is significance compared to TC83 infected cells. * $p < 0.05$, ** $p < 0.001$,*** $p < 0.0001$, \$ $p < 0.05$, \$ $p < 0.001$

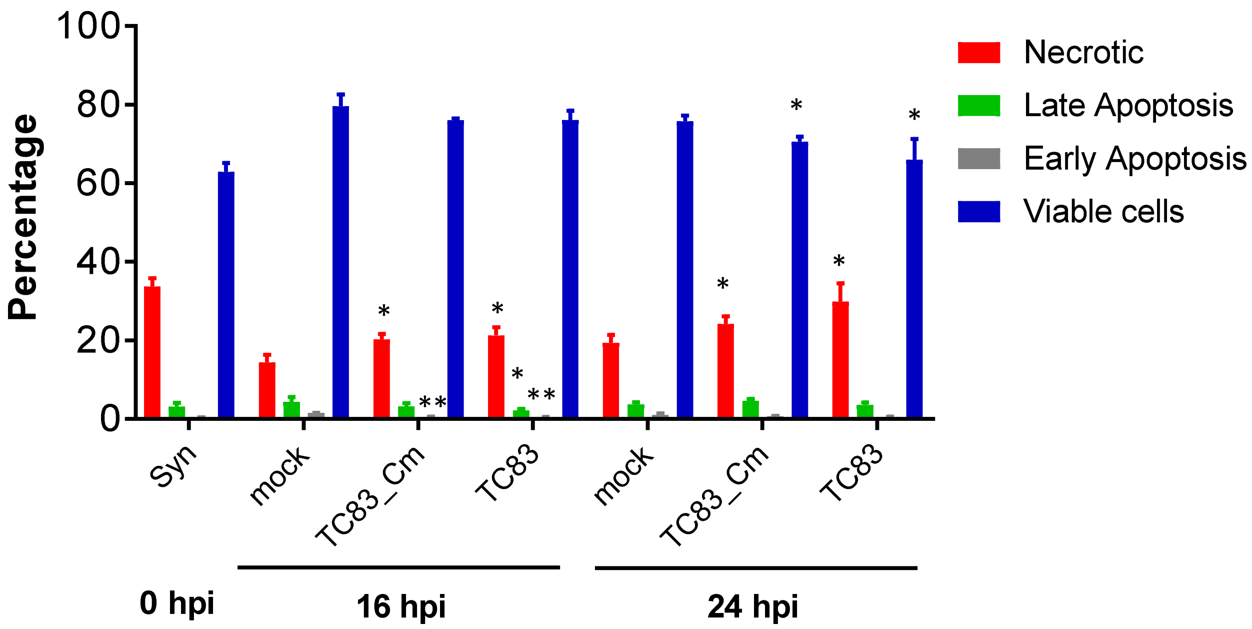

Supplementary Figure 3. U87MG cells were synchronized via serum-starvation (0.5% FBS) for 72 h. Cells were then infected (MOI 10) with wild-type TC83, TC83_Cm, or mock-infected for 1 h and then released in complete media containing 10% FBS. Cells were collected at 16 and 24 hpi and analyzed for necrotic and apoptotic cells using the eBioscience™ Annexin V Apoptosis Detection Kit eFluor™ 450 (ThermoFisher Scientific). The average of three biological replicates is displayed. *statistically significant differences from mock. * $p < 0.05$, ** $p < 0.001$,
